# Dosimetric advantages of proton therapy over conventional radiotherapy with photons in young patients and adults with low-grade glioma

**DOI:** 10.1007/s00066-016-1005-9

**Published:** 2016-06-30

**Authors:** S. B. Harrabi, N. Bougatf, A. Mohr, T. Haberer, K. Herfarth, S. E. Combs, J. Debus, S. Adeberg

**Affiliations:** 1 Heidelberg Institute of Radiation Oncology (HIRO), Im Neuenheimer Feld 400, 69120 Heidelberg, Germany; 2 Heidelberg Ion-Beam Therapy Center (HIT), Im Neuenheimer Feld 450, 69120 Heidelberg, Germany; 3 Dept. of Radiation Oncology, University Hospital Heidelberg, Im Neuenheimer Feld 400, 69120 Heidelberg, Germany; 4 Clinical Cooperation Unit Radiation Oncology, German Cancer Research Center (DKFZ), Im Neuenheimer Feld 280, 69120 Heidelberg, Germany; 5 Department of Medical Physics in Radiation Oncology, German Cancer Research Center (DKFZ), Im Neuenheimer Feld 280, 69120 Heidelberg, Germany; 6 Department of Radiation Sciences (DRS), Institute of Innovative Radiotherapy (iRT), Helmholtz Zentrum München, Ingolstädter Landstraße 1, 85764 Oberschleißheim, Germany; 7 Partner Site Munich, Deutsches Konsortium für Translationale Krebsforschung (dktk), Munich, Germany

**Keywords:** Brain tumors, Children, Neurogenesis, Quality of life, Organs at risk, Hirntumor, Kinder, Neurogenese, Lebensqualität, Risikoorgane

## Abstract

**Background and purpose:**

Low-grade glioma (LGG) is a very common brain tumor in pediatric patients typically associated with a very good prognosis. This prognosis makes it imperative that the risk of long-term treatment-related side effects be kept at an absolute minimum. Proton therapy (PRT) provides a radiation technique that has the potential to further reduce the genesis of radiogenic impairment.

**Materials and methods:**

We retrospectively assessed 74 patients with LGG who underwent PRT. Conventional three-dimensional photon and PRT plans were generated after contouring structures of neurogenesis, crucial neuronal structures, and areas susceptible to secondary malignancies. Target volume coverage was evaluated using the homogeneity index (HI) and inhomogeneity coefficient (IC). Results were compared using the Wilcoxon-signed rank test, with *p* < 0.05 being statistically significant.

**Results:**

Target volume coverage was comparable for the photon and proton plans. Overall, we could show an essential reduction in maximal, mean, and integral doses in critical neurologic structures, areas of neurogenesis, and structures of neurocognitive function. The study indicated specifically how contralaterally located structures could be spared with PRT.

**Conclusion:**

PRT is a highly conformal radiation technique offering superior dosimetric advantages over conventional radiotherapy by allowing significant dose reduction for organs at risk (OAR) that are essential for neurologic function, neurocognition, and quality of life, thus demonstrating the potential of this technique for minimizing long-term sequelae.

## Zusammenfassung

**Hintergrund und Ziel:**

Niedriggradige Gliome (LGG) zählen zu den häufigsten Hirntumoren im Kindesalter und sind üblicherweise mit einer sehr guten Prognose vergesellschaftet. Es gilt daher, das Risiko für therapieassoziierte Spätfolgen so gering wie möglich zu halten. Mit der Protonenbestrahlung steht eine Bestrahlungsmodalität zur Verfügung, mit der das Auftreten radiogener Spätfolgen im Vergleich zu konventionellen Bestrahlungstechniken weiter minimiert werden könnte.

**Material und Methoden:**

Für diese Studie wurden 74 konsekutive Patienten retrospektiv ausgewertet, die aufgrund eines LGG einer Protonenbestrahlung unterzogen wurden. Ergänzend wurden zunächst auf der Planungscomputertomographie die für neurokognitive Funktionen relevanten Zentren sowie Strukturen der Neurogenese identifiziert und konturiert; anschließend wurden korrelierende konventionelle Photonenpläne berechnet. Die Zielvolumenabdeckung wurde mithilfe des Homogenitätsindex (HI) und des Inhomogenitätskoeffizienten (IC) beurteilt und die Ergebnisse mit dem Wilcoxon-Vorzeichen-Rang-Test verglichen, wobei ein *p*-Wert < 0,05 als statistisch signifikant gewertet wurde.

**Ergebnisse:**

Die Zielvolumenabdeckung war sowohl für Photonen- als auch für Protonenpläne vergleichbar. Insgesamt konnte mit der Protonentherapie nicht nur die Integraldosis des gesunden Gehirngewebes, sondern auch die maximale und mittlere Dosisbelastung der zuvor definierten kritischen Risikoorgane deutlich gesenkt werden. Dies gilt in besonderem Maße für die dem Tumor kontralateral gelegene Hemisphäre.

**Schlussfolgerung:**

Die Protonenbestrahlung zeigt eine der konventionellen Radiotherapie mit Photonen in Hinblick auf Risikoorganschonung bei weitem überlegene dosimetrische Verteilung, mit dem großen Potential, radiogene Spätfolgen – wie die Beeinträchtigung neurologischer und neurokognitiver Funktionen und der Lebensqualität – zu minimieren.

Low-grade gliomas (LGG) are the most common type of brain tumor in children. Today, the prognosis is very good and pediatric patients are expected to become long-term survivors. It is, therefore, essential to reduce the risk of long-term side effects as much as possible.

Surgery is generally accepted as the first-line treatment if a complete resection can be achieved without major neurologic impairment. However, in many cases, only a partial resection or biopsy can be performed due to an unfavorable localization in proximity to vital structures such as the brainstem, optic system, pituitary, hypothalamus, or other areas of the brain with critical functions. Since the risk for disease progression is significantly higher in cases of subtotal compared to complete resection [1], radiotherapy plays an important role in the treatment of pediatric LGG. Radiotherapy is one of the most effective treatment alternatives, achieving high long-term control rates [2, 3]; although its use is not undisputed. Nevertheless, given the good prognosis, particular attention is paid to reduction of potential treatment-related sequelae. Younger patient age has been attributed with a higher risk of neurocognitive impairment, neurologic deficits, reduced quality of life, and secondary malignancies. The recommended lower age limit for initiation of radiotherapy differs among international protocols: European trials set the threshold at 8 years of age [4], while North American studies advise waiting until the age of 10 [5]. However, adult patients also show a tendency to develop dementia more frequently after cranial irradiation [6], which is known to be related to a significant impairment of the patient’s quality of life.

Over time, many technical advances, such as three-dimensional (3D) treatment planning, image guidance, intensity modulation, and particle therapy, have been adopted in daily routine, leading to incremental improvements in terms of conformity. Three-dimensional conformal radiotherapy (3D-CRT) is a widely available radiation technique accepted as standard [7–9]. A multitude of data is available for 3D-CRT, but long-term studies of PRT are still scarce. Along with the commissioning of new proton facilities, the use of PRT is rapidly increasing [10]. Due to its distinct biophysical properties with typically low doses in the beam entrance area and a nearly complete dose deposition in the so-called Bragg-peak, PRT is a highly conformal technique that allows steep dose gradients. As a result, excellent target coverage can be maintained without compromises due to nearby critical OAR because their tolerances are not exceeded. In view of the dosimetric superiority of PRT compared to 3D-CRT, this study was conducted to present in detail the influence on structures that are essential for neurocognitive function, in addition to specific OAR that are responsible for neurologic side effects or impairment of quality of life. In the world of radiation oncology, sparing of the hippocampus and other stem cell niches has become a topic of particular interest [11, 12]. However, there are several risk factors for the development of neurocognitive dysfunction: in addition to radiotherapy, tumor localization, the extent of resection, and concomitant chemotherapy are recognized risk factors [13]. The authors set out to determine and quantify the superiority of PRT in terms of dose distribution for the abovementioned OAR and, thereby, evaluate the potential benefit with regard to long-term radiogenic sequelae.

## Materials and methods

### Patient selection

A total of 74 patients with histologically proven LGG originally treated at the authors’ institution between 2012 and 2014 were retrospectively selected for this comparative study. All patients presented with supratentorial or infratentorial disease. Approximately half of the patients (*n* = 36; 48.6 %) were children or young adults under 30 years of age (Table 1). For treatment planning, patients were fixed using custom-made mask fixation and underwent pretherapeutic computed tomography (CT) and magnetic resonance imaging (MRI). All patients underwent 3D-PRT or intensity-modulated proton therapy (IMPT) with a median dose of 54 Gy (range 50.4–60 Gy) in 1.8 Gy per fraction (range 1.8–2.0 Gy), with a horizontal beamline using the raster scanning technique [14]. The patient collective encompassed a wide range of histological LGG subgroups among pilocytic astrocytoma (*n* = 28; 37.8 %), fibrillary astrocytoma (*n* = 22; 29.7 %), and oligodendroglioma (*n* = 6; 8.1 %). Median age of patients with pilocytic astrocytoma was 16.2 years (2.0–53.3 years) and this was 36.3 years (5.9–64.2 years) for other LGG.

**Table 1:** Patient characteristics

Cofactors	All *n* = 74 (%)
Gender	
Male	39 (52.7)
Female	35 (47.3)
Median age in years (range)	31.2 (2.0–64.2)
Surgical resection	43 (58.1)
Gross total resection	11 (14.9)
Subtotal resection	31 (41.9)
Resection status unclear	2 (2.7)
Biopsy	30 (40.5)
Median PTV volume in ml (range)	185.2 (11.8–709.6)
Median total dose in Gy (range)	54.0 (50.4–60)
Median single dose in Gy (range)	1.8 (1.8–2.0)

*PTV* planning target volume

### Contouring and treatment planning

Contouring was performed on the patients’ original treatment planning CT scans and fused with the pretherapeutic MRI using contrast-enhanced T2 fluid-attenuated inversion recovery (FLAIR) imaging. The initial gross tumor volume (GTV) was defined as the hyperintense low-grade tumor mass, surgical resection cavity, and perifocal edema on T2-FLAIR. A safety margin of up to 1 cm was added for the clinical target volume (CTV) to account for microscopic spread. All OAR were contoured using coregistered T1-weighted postcontrast MRI on axial views. In the authors’ study group, at the time of initial treatment planning and delivery, the additional OAR were not explicitly contoured, monitored, or used as avoidance structures for either treatment technique. Contouring of the initial and additional OAR and the treatment volume definition for photon and particle therapy planning was performed using the Siemens Dosimetrist and Oncologist software (Siemens, Erlangen, Germany). Photon RT and PRT re-planning were performed on the original planning CT datasets and dose recalculation was done using the initial planning parameters. Furthermore, additional crucial cerebral structures for neurogenesis, secondary malignancies, and neuronal functions were contoured retrospectively. To allocate the laterality, we determined the tumor key area regardless of bilateral tumor growth. Ipsilateral (IL) and contralateral (CL) subventricular zones (SVZ) were contoured as a 5-mm margin lateral to the lateral ventricles as previously described [15, 16]. The hippocampus and amygdala were contoured according to previously published guidelines [17]. The hypothalamus and thalamus were contoured in accordance with previously published contouring landmarks [18, 19]. Brain structures were contoured including supraentorial and infratentorial brain areas. The brainstem comprised the midbrain, pons, and medulla oblongata. The pituitary gland, cochlear IL and CL, optic nerves, and chiasm were identified and contoured following previously published recommendations [20]. Treatment planning was performed by a single experienced radiation therapist using the Oncentra MasterPlan® (Nucletron, Columbia, SC, USA) planning system, version 4.5, with a collapsed cone algorithm for 3D photon treatment planning. In 3D-CRT, beam directions were carefully selected and consisted of four to five coplanar and non-coplanar fields in the majority of cases and, if necessary, subfields, using a field-in-field (FIF) technique. For all patients, 6‑MV photons were used. Treatment planning for proton therapy was performed using the treatment planning system Syngo PT Planning (Siemens). For the plan comparison, ion beams were applied using a horizontal beam or the gantry. Two to three coplanar or non-coplanar beams were used for the particle therapy treatment. The gantry rotation was not restricted in the coplanar and non-coplanar settings. The pencil beams chosen for the PRT typically had a lateral full width at half maximum (FWHM) of 10 mm. The treatment table position was restricted to between 10 and 170 degrees to avoid collisions of the horizontal beam nozzle with the table. To assure comparability, the same target volumes and OAR were used for particle and photon plans. Tolerance levels for the OAR were based on the work of QUANTEC [21–25]. Planning target volume (PTV) coverage of ≥ 95 % of the prescribed dose was required and in all patients, at the time of treatment planning and delivery, the additional OAR were monitored but not considered an avoidance structure in either radiotherapy modality.

### Treatment plan evaluation

Qualitative and quantitative dose evaluations were conducted for both radiotherapy modalities. Dose–volume histograms (DVHs) were constructed for all volumes, and dose parameters were extracted to check for proper target volume coverage and to assure compliance with the OAR dose constraints. PTV coverage was assessed utilizing measurement of the volume receiving ≥ 90 %, ≥ 95 %, and ≥ 100 % of the prescribed relative doses (in %). Confirmation of PTV dose distribution was evaluated by calculating HI and IC.

$$\textit{Homogeneity index} (HI)=\frac{\text{D}_{5}-\text{D}_{95}}{\text{Dp}}x100$$

$$\textit{Inhomogeneity coefficient} (IC)=\frac{\text{D}_\text{max}-\text{D}_\text{min}}{\text{D}_\text{mean}}$$

D_5_ and D_95_ are the minimum doses in 5 % and 95 % of the PTV, respectively, and D_p_ is the prescribed dose in the PTV. The ideal HI value is zero, where D_5_ equals D_95_ [26]. The IC assesses the distribution variance of the PTV dose, where higher values indicate greater variability [27]. D_max_ and D_min_ represent the maximum and minimum doses in the PTV, respectively, and D_mean_ equals the average PTV dose.

$$\textit{Integral dose} (ID)= \sum\limits_{i} \text{D}_\text{mean}\times V_i$$

The integral dose (ID) is defined as the sum of the mean dose multiplied by the volume if the voxels are assumed to be the same size and the organ is hypothesized to have a uniform density. The ID also represents the area under the DVH curve at all dose levels [28] and allows evaluation of the lower dose spread compared to conventional measurements. D_mean_ equals the average dose of the target volume or OAR, and V_i_ is defined as the structure volume in ml. The simplified formula was used in this analysis: D_mean_ × volume.

### Data management and automatic dose–volume analysis

All PRT data and additional treatment information were available in the central research database of the authors’ department, which functioned as the central data source like previously described [29]. Project-specific OAR re-contouring and 3D-CRT data were additionally imported into the central research database after re-planning. Dose–volume analysis was performed automatically on a central analysis platform directly connected to the central research database like previously described [30]. A workflow was designed to analyze the radiotherapeutic imaging data (RT data) of all patients with the abovementioned re-contouring. First, RT data was retrieved from the central research database and preprocessed for analysis. During analysis, dose statistics and DVHs were calculated automatically. All results were written into the central storage of the analysis platform. Finally, results for all patients were summarized in a single result file for further statistical analysis.

### Ethics

The study was approved by the Ethics Committee, University of Heidelberg (no. S‑056/2015).

### Statistical analysis

Statistical analysis was carried out with SigmaPlot™ (Systat Software GmbH, Erkrath, Germany) software. The Wilcoxon signed-rank test was applied for analysis with corresponding two-sided 95 % confidence intervals. A *p*-value < 0.05 was considered statistically significant.

## Results

### PTV coverage

**Fig. 1:**
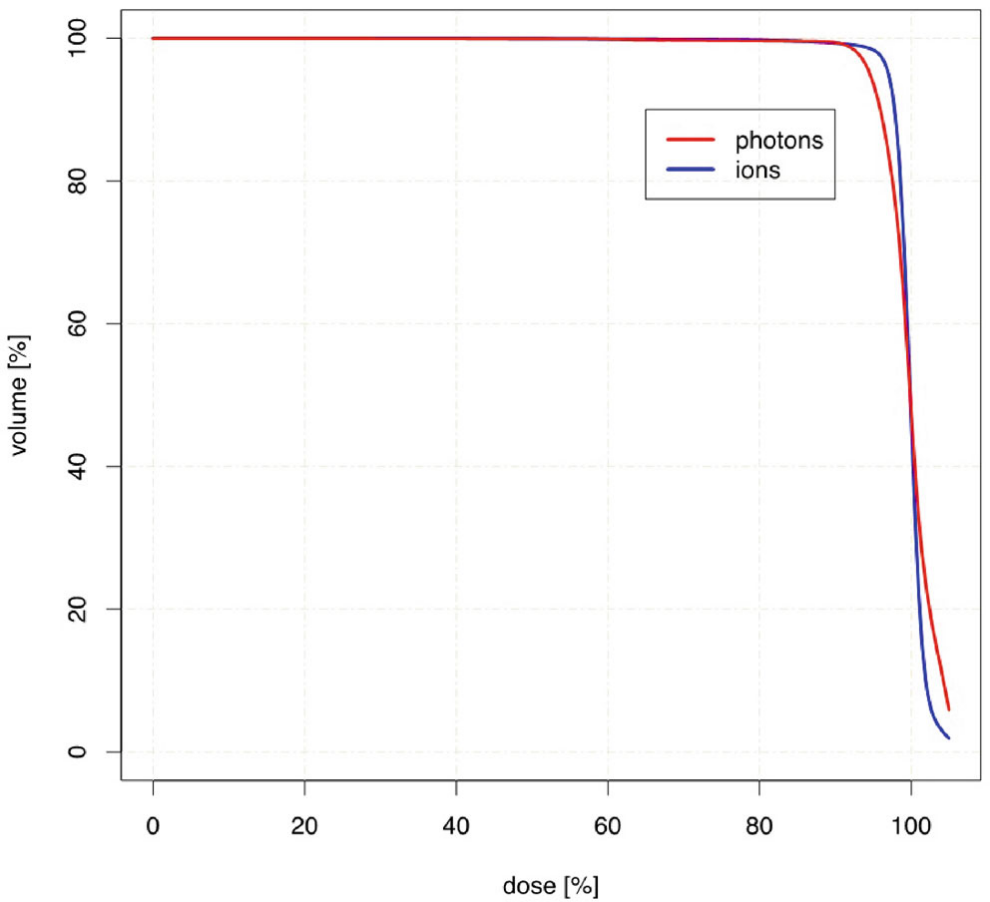
Cumulative dose–volume histogram DVH (*n* = 74) comparing planning target coverage for both proton beam therapy (*PRT*) and three-dimensional conventional radiotherapy (*3D-CRT*)

Target volume coverage was comparable in both treatment modalities. No statistically significant difference could be detected regarding V_90__%_ and V_95__%_ (percentage of PTV receiving a minimum of 90 and 95 % of the prescribed dose, respectively). Similar findings for values can be identified by comparing the HI and IC (Table 2). Although not significant, when comparing the cumulative DVH for PTV, slightly better target coverage as well as a lower maximum dose for protons can be observed (Fig. 1).

**Table 2:** Comparison of target volume coverage

	3D-CRT	PRT	*p*-value
V_90__% _(in %)	96.10 ± 3.56	97.54 ± 3.26	0.238
V_95__% _(in %)	94.62 ± 5.95	96.36 ± 5.26	0.361
V_100__% _(in %)	85.12 ± 12.08	85.75 ± 9.35	0.751
D_max_ (in %)	105.81 ± 2.82	106.56 ± 2.45	0.324
D_mean_ (in %)	99.66 ± 1.89	99.78 ± 1.39	0.979
D_min_ (in %)	85.23 ± 12.08	84.90 ± 12.21	0.969
HI (in %)	8.85 ± 5.62	5.84 ± 5.42	0.001*
IC (in %)	0.21 ± 0.13	0.22 ± 0.13	0.694

Values are given as mean values with standard deviations

*Indicates a statistically significant *p*-value

*3D-CRT* three-dimensional conventional radiotherapy, *PRT* proton beam therapy, *V*_90__%_ percentage of planning target volume (PTV) that receives a minimum of 90 % of the prescribed dose, *V*_95__%_ percentage of PTV that receives a minimum of 95 % of the prescribed dose, *V*_100__%_ percentage of PTV that receives a minimum of 100 % of the prescribed dose, *D*_max_ maximum dose to the PTV, *D*_mean_ average dose to the PTV, *D*_min_ minimum dose to the PTV, *HI *homogeneity index: (D_5__%_–D_95__%_)/prescribed dose × 100, *IC *inhomogeneity coefficient: (D_max_–D_min_)/D_mean_

### Sparing organs at risk

#### Critical organs of the central nervous system

**Fig. 2:**
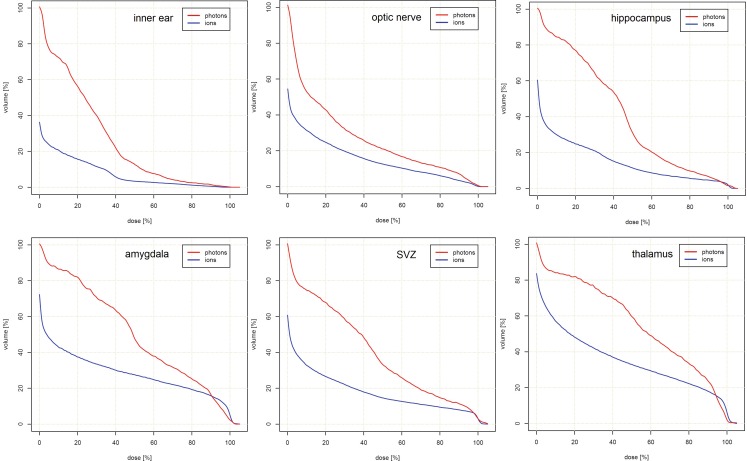
Cumulative dose–volume histograms for contralateral organs at risk showing a significant dose reduction for proton beam therapy compared to three-dimensional conventional radiotherapy. *SVZ* subventricular zone

**Fig. 3:**
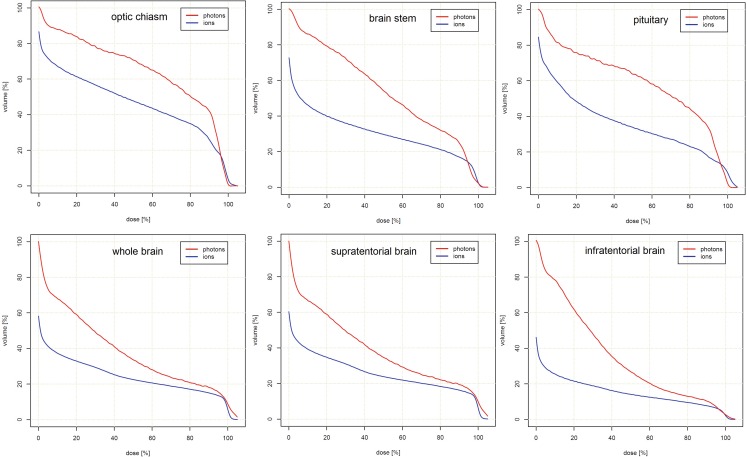
Cumulative dose–volume histograms for unifocal organs at risk showing a significant dose reduction for proton beam therapy compared to three-dimensional conventional radiotherapy

**Fig. 4:**
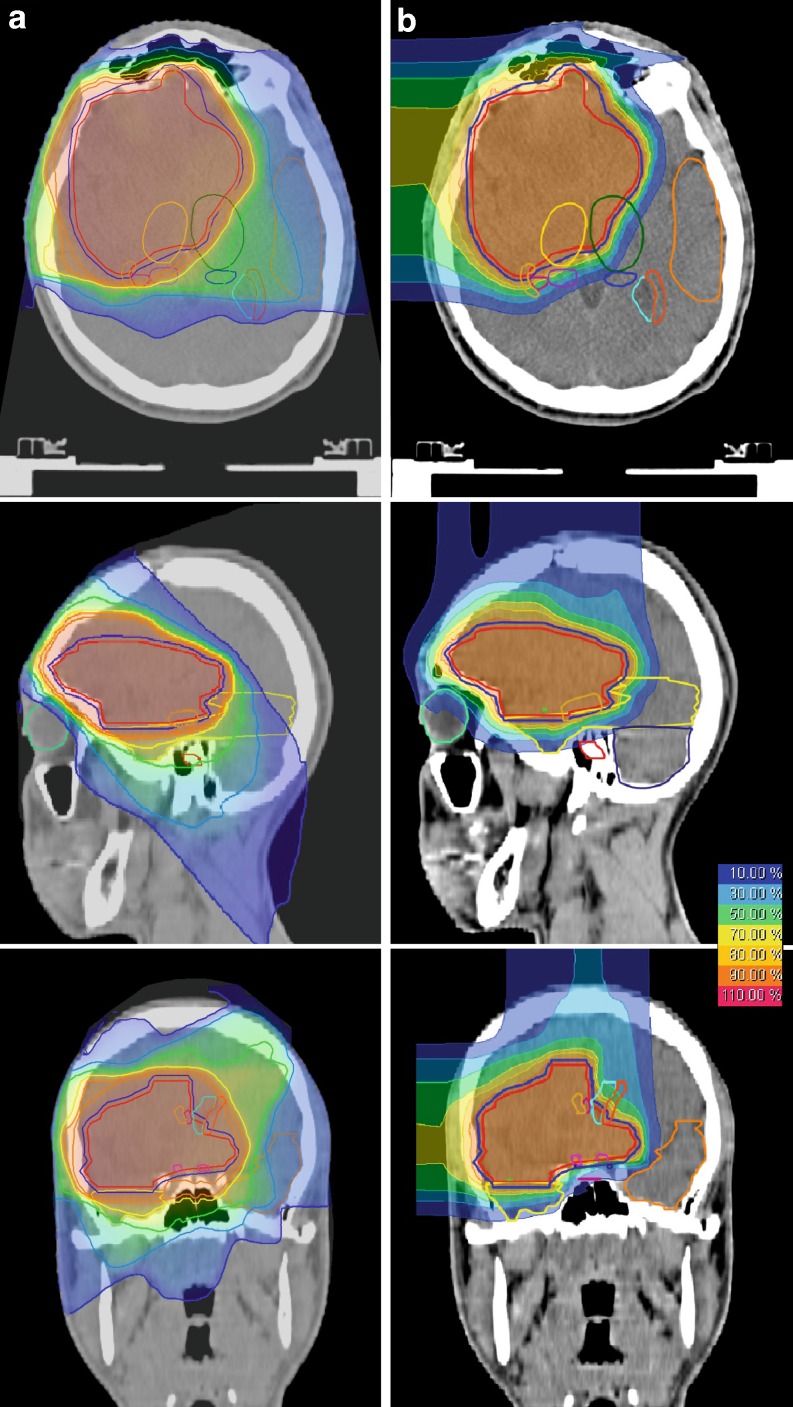
Comparison of dose distribution for a patient with low grade glioma. **a** Three-dimensional conventional radiotherapy plan, **b** proton beam therapy plan. CTV is delineated in *red*, the corresponding planning target volume in *blue*. The potential for dose reduction is especially eminent at the contralateral site

Dose-limiting OAR with limiting tolerance doses and regions at risk of secondary malignancies were assessed to compare both treatment modalities (Table 3 and Fig. 4). PRT allowed for significant sparing of critical IL and CL auditory organs. Here, the D_max_ and ID doses were decreased by 21.6 and 38.1 % IL, and 63.7 and 74.1 % CL, respectively. Similar results were seen for the optical system, where the dose to the optic nerve (ON) CL and optic chiasm were decreased by 35.6 % (D_max_), 44.0 % (D_mean_), and 40.7 % (ID); and 12.7 % (D_max_), 30.0 % (D_mean_), and 25.2 % (ID), respectively. Findings for the IL ON did not reach statistical significance. PRT reduced the D_max_ and ID to the centrally located pituitary gland by 24.6 and 37.8 %, respectively. Normal brain tissue, as a potential risk area for secondary malignancies, could be spared significantly. Here, the ID to supratentorial and infratentorial brain areas could be reduced by 33.7 and 48.3 %, respectively (each < 0.001). Similar decreases in D_mean_ (45.6 and 38.0 %) and ID (47.1 and 37.4 %) followed for the brainstem and whole brain, respectively (each <0.001). Cumulative DVHs are shown in Figs. 2 and 3.

**Table 3:** Dose comparison of organs at risk

Organ at risk		3D-CRT (relative dose in % ± SD)	PRT (relative dose in % ± SD)	3D-CRT vs. PRT (difference in %)	*p*-value
Optic nerve ipsilateral	D_max_	64.4 ± 35.5	56.2 ± 39.0	−12.8	0.165
	D_mean_	38.4 ± 28.3	33.0 ± 28.5	−14.0	<0.001*
	Mean ID	62.8 ± 61.0	54.7 ± 59.5	−12.9	0.480
Optic nerve contralateral	D_max_	56.5 ± 33.9	36.4 ± 36.7	−35.6	<0.001*
	D_mean_	26.4 ± 22.0	14.8 ± 20.1	−44.0	<0.001*
	Mean ID	43.2 ± 43.7	25.6 ± 44.7	−40.7	0.008*
Inner ear ipsilateral	D_max_	56.8 ± 33.4	44.5 ± 37.6	−21.6	0.015*
	D_mean_	43.4 ± 29.5	26.3 ± 26.9	−39.3	<0.001*
	Mean ID	61.6 ± 43.1	38.1 ± 41.4	−38.1	<0.001*
Inner ear contralateral	D_max_	34.6 ± 23.1	12.5 ± 22.8	−63.7	<0.001*
	D_mean_	24.9 ± 18.8	7.2 ± 15.3	−71.0	<0.001*
	Mean ID	35.7 ± 31.1	9.2 ± 19.7	−74.1	<0.001*
Optic chiasm	D_max_	76.6 ± 28.8	66.8 ± 35.7	−12.7	0.033*
	D_mean_	63.5 ± 29.8	44.5 ± 33.8	−30.0	<0.001*
	Mean ID	146.1 ± 96.4	109.2 ± 103.2	−25.2	0.017*
Thalamus ipsilateral	D_max_	82.4 ± 27.0	83.5 ± 26.6	1.3	0.059
	D_mean_	66.7 ± 31.3	58.8 ± 32.8	−11.9	<0.001*
	Mean ID	498.0 ± 266.1	433.1 ± 270.5	−13.0	0.025*
Thalamus contralateral	D_max_	76.2 ± 27.9	69.3 ± 35.2	−9.0	0.598
	D_mean_	54.5 ± 27.7	32.9 ± 29.1	−39.7	<0.001*
	Mean ID	419.4 ± 241.6	260.5 ± 243.9	−37.9	<0.001*
SVZ ipsilateral	D_max_	88.9 ± 19.0	86.3 ± 22.1	−3.0	0.301
	D_mean_	57.9 ± 30.8	50.5 ± 31.2	−12.9	<0.001*
	Mean ID	765.5 ± 552.4	731.9 ± 639.9	−4.4	0.069
SVZ contralateral	D_max_	75.5 ± 23.7	61.4 ± 36.0	−18.7	0.006*
	D_mean_	38.2 ± 21.9	16.8 ± 18.7	−56.1	<0.001*
	Mean ID	531.7 ± 342.4	246.9 ± 350.6	−53.6	<0.001*
Hypothalamus	D_max_	78.9 ± 27.1	72.5 ± 35.4	−8.2	0.494
	D_mean_	67.6 ± 29.6	50.4 ± 35.2	−25.5	<0.001*
	Mean ID	192.9 ± 107.2	144.2 ± 117.2	−25.2	<0.001*
Hippocampus ipsilateral	D_max_	84.6 ± 22.0	80.0 ± 29.5	−5.5	0.610
	D_mean_	64.0 ± 30.5	54.2 ± 35.9	−15.3	<0.001*
	Mean ID	391.7 ± 207.8	327.2 ± 229.8	−16.5	0.060
Hippocampus contralateral	D_max_	64.7 ± 24.7	40.6 ± 39.2	−37.2	<0.001*
	D_mean_	39.1 ± 21.6	13.9 ± 21.6	−64.5	<0.001*
	Mean ID	248.6 ± 149.8	92.8 ± 156.7	−62.7	<0.001*
Amygdala ipsilateral	D_max_	77.0 ± 29.3	70.0 ± 36.5	−9.2	0.128
	D_mean_	69.1 ± 32.3	59.9 ± 38.1	−13.3	<0.001*
	Mean ID	52.0 ± 39.5	48.0 ± 47.8	−7.7	0.075
Amygdala contralateral	D_max_	60.4 ± 30.0	39.2 ± 41.0	−35.0	<0.001*
	D_mean_	49.5 ± 28.5	26.6 ± 34.0	−46.3	<0.001*
	Mean ID	41.0 ± 35.5	25.4 ± 41.9	−37.9	<0.001*
Lateral ventricle ipsilateral	D_max_	89.1 ± 18.6	88.0 ± 20.8	−1.3	0.841
	D_mean_	59.0 ± 30.2	50.0 ± 29.5	−15.2	<0.001*
	Mean ID	1085.9 ± 1306.3	976.6 ± 1009.1	−10.1	0.035*
Lateral ventricle contralateral	D_max_	81.3 ± 22.4	75.9 ± 30.0	−6.7	0.315
	D_mean_	45.6 ± 24.9	25.0 ± 22.9	−45.3	<0.001*
	Mean ID	896.3 ± 833.3	511.3 ± 687.4	−42.9	<0.001*
Supratentorial	D_max_	103.4 ± 10.4	103.7 ± 12.8	+0.3	0.386
	D_mean_	37.8 ± 19.6	24.5 ± 14.8	−35.2	<0.001*
	Mean ID	45154.0 ± 24207.3	29958.7 ± 19079.9	−33.7	<0.001*
Infratentrorial	D_max_	90.5 ± 25.7	88.1 ± 32.8	−2.7	0.447
	D_mean_	33.5 ± 18.7	14.3 ± 19.0	−57.5	<0.001*
	Mean ID	7388.1 ± 6400.3	3818.6 ± 10575.3	−48.3	<0.001*
Pituitary gland	D_max_	65.3 ± 33.8	49.2 ± 38.2	−24.6	0.003*
	D_mean_	57.8 ± 33.4	34.1 ± 35.1	−40.9	<0.001*
	Mean ID	34.7 ± 27.4	21.6 ± 25.8	−37.8	<0.001*
Brain stem	D_max_	86.4 ± 16.7	79.4 ± 27.5	−8.2	0.153
	D_mean_	51.7 ± 23.1	28.1 ± 26.3	−45.6	<0.001*
	Mean ID	1467.4 ± 947.2	775.9 ± 761.2	−47.1	<0.001*
Brain	D_max_	105.8 ± 2.9	106.4 ± 3.1	+0.6	0.447
	D_mean_	37.2 ± 17.2	23.1 ± 12.3	−38.0	<0.001*
	Mean ID	52898.6 ± 24823.9	33100.1 ± 18828.2	−37.4	<0.001*

*Indicates a statistically significant *p*-value.

*3D-CRT* three-dimensional conventional radiotherapy, *PRT* proton beam therapy, *D*_max_ maximum dose to the planning target volume (PTV), *D*_mean_ average dose to the PTV, *D*_min_ minimum dose to the PTV, *ID* integral dose, *SVZ* subventricular zone, *SD* standard deviation

#### Critical organs of neurogenesis

It has been shown that neuronal stem cells—initiating cells for neurogenesis even in adult individuals—are extremely sensitive [31] and show diverse recovery behaviors after exposure to ionizing radiation [32]. Sparing of the IL SVZ was only significant for D_mean_ (−12.9 %). However, D_max_ and ID of the CL SVZ could be reduced by 18.7 % (<0.001) and 53.6 % (<0.001), respectively, using PRT. The second anatomic region that has been shown to harbor neuronal stem cells is the dentate gyrus in the hippocampal formation. For the CL hippocampus, doses could be reduced by D_max_: 37.2 %, D_mean_: 64.5 %, and ID: 62.7 % (each <0.001). For the IL hippocampus, only the D_mean_ to the hippocampus could be decreased significantly at 15.3 % by applying a PRT plan (< 0.001).

#### Critical structures of neurocognitive functions

D_max_ (35.0 %), D_mean_ (46.3 %), and ID (37.9 %) to the CL amygdala could be spared using proton plans. Analogous to other IL structures, the IL amygdala had reductions of D_mean_ by only 13.3 % (<0.001). Again, sparing of the CL thalamus was superior to sparing of the IL thalamus when using PRT plans. Relative decreases of D_mean_: 39.7 % and ID: 37.9 % (each <0.001) in the CL thalamus and D_mean_: 13.0 % and ID: 11.9 % (*p* = 0.025 and *p* < 0.001, respectively) in the IL thalamus could be achieved.

### Pilocytic astrocytoma versus other LGG

When assigning the patient collective further into pilocytic astrocytoma (*n* = 28) and other LGG (*n* = 46), the latter could be spared more effectively with PRT than the former. In detail, in the CL hippocampus (D_max_: −97.6 % vs. −60.5 %, D_mean_: −79.2 % vs. −3.0 %, ID: −97.8 % vs. −61.1 %), CL amygdala (D_max_: −96.2 % vs. −33.3 %, ID: −95.6 % vs. −36.7 %), brainstem (D_max_: −77.2 % vs. −23.9 %, ID: −76.7 % vs. −23.3 %), pituitary gland (D_mean_: −45.5 % vs. −3.6 %, ID: −70.5 % vs. −12.6 %) and optic chiasm (D_max_: −37.5 % vs. −16.6 %), the dose reduction with PRT was more pronounced for non-pilocytic astrocytoma. Only in the IL hippocampus (D_max_: +4.5 % vs. −20.8 %) and IL inner ear (D_mean_: −6.6 % vs. −27.3 %) did PRT decrease the dose in a more pronounced manner in pilocytic astrocytoma.

## Discussion

These data present an overwhelming dosimetric advantage of PRT over 3D-CRT in terms of sparing not only stem cell niches, but also nearly any other OAR. In addition to the significant reduction of mean dose and the ID bilaterally, particularly on the CL side, the maximum dose could be lowered. PRT offers distinct biophysical advantages over conventional 3D-CRT. However, to what extent this will have a clinical impact remains to be proven by long-term observations. Particular attention must be paid to structures that are considered essential for neurocognitive functions, such as the hippocampus or the SVZ.

There are several reports of treatment planning comparisons for various entities. Boehling et al., for example, investigated the dose distribution in 10 representative cases of pediatric craniopharyngioma for nearby critical structures when using PRT instead of intensity modulated radiotherapy with photons. Their study showed a reduction of the ID received by the hippocampus of up to 51 % and up to 57 % for the SVZ [33], whereby intensity modulated PRT had the largest potential for relative dose reduction. Another study published by Fuss et al. performed a dosimetric comparison for optic pathway glioma focusing on the saving potential for the CL ON, showing that PRT has the potential to nearly halve the dose received (−47 %) [34]. A decrease in ID was also noted for the chiasm (−11 %) and the pituitary (−13 %). Likewise, dosimetric superiority for the whole brain, temporal lobes, chiasm, and cochlea could be demonstrated in both supratentorial and infratentorial locations [10, 35, 36]. The ID represents a valuable option for considering OAR volumes in the dosimetric assessment. Here the ID should offer objective values, which allow for improved evaluation of lower dose spreads compared to D_mean_ or median dose. However, even the ID is not able to predict normal toxicity complication probability (NTCP) without correlation of clinical long-term toxicity data. All of these reports are in accordance with the presented findings that there is no difference between photon and proton plans with regard to target coverage, all the more, however, for the CL OAR, particularly when using intensity modulated PRT instead of 3D-PRT.

It is well known that the risk of occurrence and the severity of radiation-related impairment of neurocognitive function are correlated with both the dose and the irradiated volume of critical structures, such as the supratentorial brain in general, or the hippocampus in particular. Especially in children, a decrease in intelligence quotient (IQ), processing speed, and fine motor skills has been reported [37–39]. Our particular focus was on the exposure of structures considered crucial for neurocognitive performance, such as the hippocampus, SVZ, amygdala, and thalamus. In contrast to a general assessment of dose distribution (whole brain, supratentorial, infratentorial, temporal lobe), a more detailed analysis substantiated by clinical parameters could lead to a better understanding and risk assessment for the occurrence of neurocognitive impairment. Merchant et al. showed that the dose reduction achieved by PRT has the potential to mitigate neurocognitive impairment. The authors collected dosimetric information for 40 patients with different types of childhood brain tumors and calculated the estimated decline in their full scale IQs using dose-dependent cognitive effect models [40]. The reason for radiation-induced neurocognitive impairment is most likely multifactorial [13, 39]; however, there is growing evidence that supports the idea that neural progenitor cells (NPCs) in stem cell niches play an important role. The hippocampus and the SVZ are known areas of origin for NPCs [11, 12]. Although their role has not yet been fully elucidated, it is hypothesized that their capability for self-renewal and injury repair is of central importance to the genesis of long-term neurocognitive effects [41, 42]. To counteract a radiogenic impairment of their recovery potential, every effort should be made to decrease the dose received by the NPCs. While there is substantiated data for the correlation between dose and hippocampus, the role of the SVZ is disputed more vigorously [43]. One of the reasons is attributed to its potential to contribute to tumor propagation [44].

As the current findings show, inter alia, PRT is an excellent treatment option that does not compromise target coverage. Furthermore, these data underline that sparing of cerebral OAR is more pronounced in the group of LGG compared to pilocytic astrocytoma, which arise mainly from midline structures. Here, PRT allows for improved sparing of centrally localized neuronal structures like the hippocampus, optic chiasm, brainstem, and pituitary gland. Preliminary results from the prospective Radiation Therapy Oncology Group (RTOG 0933) described the effect of hippocampal sparing during whole brain irradiation and concluded that decreased short-term memory impairment compared to historical controls is attributed to sparing of stem cell niches [45]. Whether, and how, these preliminary results will be reflected in a clinically relevant decrease of treatment-related long-term toxicity remains to be seen and, of course, substantiated by long-term results.

Although the present findings provide strong evidence in favor of PRT, their limitations should be considered. First, following the recommendation of the German Society of Radiation Oncology (DEGRO), PRT should be used subject to disease, localization and availability of patients with LGG, as they are particularly appropriate candidates. LGG is the most common diagnosis of central nervous system malignancy in pediatric patients and the use of radiotherapy must inevitably be considered, especially in patients presenting with an unfavorable or irresectable tumor location. In keeping with that recommendation, this study was performed retrospectively. Second, the analysis compared photons to protons in general, and this does not demonstrate the full potential of each technique. A more detailed evaluation of the role of intensity modulation for both photons and protons is planned as the next step. Equally interesting would be the possibility of taking the sensitive OAR into consideration for treatment planning initially and also consideration of whether there is an additional potential for sparing OAR with protons, as is well-known from earlier planning studies with photons based on the motto that “seeing is saving.”

The strength of this study is its large number of patients with proven LGG. To our knowledge, this is the largest case series of LGG patients, providing valuable dosimetric information because of its homogenous distribution to all lobes of the brain. To minimize interobserver variability, all OAR and all treatment plans were contoured/performed by the same experienced radiation oncologist. In addition, automated analysis of treatment-related data using a central research database and the task-customized workflow minimized potential transmission or inadvertent errors. The information obtained by the presented analysis is not only helpful for identifying patients who would potentially benefit from PRT, but also provides important arguments in interdisciplinary discussions about why the effort should be made to expand access to PRT.

However, as previously stated, the availability of proton centers remains limited. This is why the authors’ goal is to acquire new data and correlate dosimetric information with functional outcomes, potentially providing further selection criteria for patients who would benefit significantly from PRT.

## Conclusion

The dose distribution of PRT is significantly superior when compared to conventional radiotherapy, particularly with regard to OAR that are considered essential for neurologic function and neurocognition, or which play an important role in terms of quality of life. PRT might hereby lead to reduced treatment-related side effects.
